# Supplementary material for: Content-rich biological network constructed by mining PubMed abstracts
Source: BMC Bioinformatics. 2004 Oct 8;5:147. doi: 10.1186/1471-2105-5-147 (PMC528731; doi:10.1186/1471-2105-5-147)
Supplement: Additional File 2 — The original results of the above study (non-essential files are deleted to keep the file size under the limit set by BMC bioinformatics). [file 1471-2105-5-147-S2.bz2 › chilibotAdditionalFile2/dip05/411ID7673243E10074/html/BMP4_BMPR1A.html]

 


 **BMP4** and **BMPR1A** 
  
Found 15 abstracts in PubMed, retrieved 05.  
 

 What does Google say? 
 PDF only 
| .edu only 

---

**Interactive relationship** (e.g. stimulation, inhibition, etc)

**Neutral relationship**- BMP 4  [ **BMP4** ]  and GDF 5 are known to bind to activin receptor like kinase 3  [ **BMPR1A** ]  ALK 3  [ **BMPR1A** ]  and or ALK 6 also termed BMP type IA and type IB receptors, respectively.  Ref: 11282024 J Cell Sci, 2001

**Non-interactive relationship** (e.g. studied together, co-existance, homology, etc.)

- Developmental expression of  **BMP4**  ALK3  [ **BMPR1A** ]  SMAD5 signaling pathway in the mouse testis a potential role of  **BMP4**  in spermatogonia differentiation.  Ref: 12857787 J Cell Sci, 2003
- In the present study, we examine the effect of  **BMP4**  on the development of metanephric and periureteral mesenchymal cells, which express the BMP type I receptor gene,  **Bmpr1a**  Alk3  [ **BMPR1A** ] .  Ref: 12631064 Kidney Int, 2003
